# Factors in Parenting Stress in Young Patients With Breast Cancer and Implications for Children’s Emotional Development: The PSYCHE Study

**DOI:** 10.1001/jamanetworkopen.2023.44835

**Published:** 2023-11-28

**Authors:** Yungil Shin, Harin Kim, Taeyeop Lee, Seonok Kim, Sae Byul Lee, Jisun Kim, Il Yong Chung, Beom Seok Ko, Jong Won Lee, Byung Ho Son, Sei Hyun Ahn, Hyo-Won Kim, Hee Jeong Kim

**Affiliations:** 1 Department of Surgery, University of Ulsan College of Medicine, Asan Medical Center, Seoul, South Korea; 2 Department of Neuropsychiatry, Chamjoeun Hospital, Gyeonggi-do, Korea; 3 Department of Psychiatry, University of Ulsan College of Medicine, Asan Medical Center, Seoul, South Korea; 4 Department of Clinical Epidemiology and Biostatistics, University of Ulsan College of Medicine, Asan Medical Center, Seoul, South Korea

## Abstract

**Importance:**

The number of patients with breast cancer who have children is substantial. However, the emotional burden of this disease and its implication for childhood development remain largely unknown.

**Objective:**

To investigate the clinical factors in parenting stress in mothers with breast cancer and the association of maternal depression and parenting stress with their children’s emotional development.

**Design, Setting, and Participants:**

This cross-sectional study involved a survey of females with stage 0 to 3 breast cancer and was conducted from June 2020 to April 2021 in Seoul, South Korea. Participants were aged 20 to 45 years and completed the Center for Epidemiologic Studies Depression–Revised scale and basic questionnaires on demographic and clinical characteristics. Participants with children completed the Korean Parenting Stress Index Short Form (K-PSI-SF), Child Behavior Checklist, Junior Temperament and Character Inventory, and Children’s Sleep Habits Questionnaire.

**Exposure:**

Having children in patients with breast cancer.

**Main Outcomes and Measures:**

Multivariable logistic and linear regression analyses were performed to investigate the association between maternal depression and parenting stress.

**Results:**

A total of 699 females (mean [SD] age, 39.6 [4.6] years) were included, of whom 499 had children (mean [SD] age of children, 8.0 [2.7] years). Depression was more common in patients with children (odds ratio [OR], 2.25; 95% CI, 1.01-5.05) and patients who had gonadotropin-releasing hormone treatment (OR, 1.68; 95% CI, 1.15-2.44). Disease duration was inversely associated with depression (OR, 0.85; 95% CI, 0.76-0.96). Cancer-related factors were not associated with the K-PSI-SF score. Having children aged 6 years or older (β = 3.09; 95% CI, 0.19-5.99); being the sole primary caregiver (β = −3.43; 95% CI, −5.87 to −0.99); and reporting certain temperament (eg, novelty seeking: β = 0.58; 95% CI, 0.46-0.71), emotional problems (eg, anxious/depressed: β = 8.09; 95% CI, 3.34-12.83), and sleeping pattern (eg, bedtime resistance: β = 0.57; 95% CI, 0.15-0.99) subscale scores in their children were associated with parenting stress. Depression and parenting stress were correlated (β = 0.56; 95% CI, 0.45-0.66; *P* < .001). The emotional challenges encountered by children of mothers with breast cancer were not significantly different from reference values.

**Conclusions and Relevance:**

This study found that in patients with breast cancer, child-related factors and depression were significantly associated with parenting stress, but breast cancer–related factors were not correlated. The findings suggest that mothers with breast cancer are susceptible to both depression and parenting stress and that tailored counseling and support are needed.

## Key Points

**Question:**

What factors contribute to parenting stress among mothers with breast cancer, and does maternal breast cancer affect children’s emotional development?

**Findings:**

In this cross-sectional study of 699 females with breast cancer, of whom 499 were mothers, having children, a shorter disease duration, and gonadotropin-releasing hormone treatment were risk factors for depression. Child-related factors were associated with parenting stress, but clinical and treatment–related factors were not correlated.

**Meaning:**

Findings of this study suggest that mothers with breast cancer are susceptible to both depression and parenting stress and that counseling and support tailored to this patient group are imperative.

## Teaser

This cross-sectional study examines the clinical factors in parenting stress in mothers with breast cancer and the association of maternal depression and parenting stress with their children's emotional development.

## Introduction

According to the 2019 National Cancer Registration, breast cancer is the most common cancer among females in South Korea.^1^ Furthermore, data from the National Cancer Institute have shown that over 30% of patients with breast cancer younger than 54 years have children younger than 18 years.^2^ In East Asia, the proportion of middle-aged patients with breast cancer is higher than that in the US.^3^ Consequently, a substantial number of these patients are expected to be the primary caregivers of their children while managing their disease. For mothers with breast cancer, parenting can be challenging, especially during hospitalization or chemotherapy, which can place considerable psychological distress on these patients. Additionally, the children are required to adjust to their mother’s disease and related absence and may also experience anxiety and worries about losing their mother.^4,5^ Therefore, children may be vulnerable to disrupted emotional development.

Previous research has suggested that maternal breast cancer affects children’s emotional development.^6,7,8,9^ Wellisch et al^6^ reported that daughters of mothers with breast cancer had psychological symptoms that were comparable with those of daughters with mothers without a history of the disease, whereas Armsden and Lewis^7^ found that children of patients with breast cancer scored higher on behavioral adjustment and lower on self-esteem compared with a control group. In contrast, Howes and colleagues^8^ reported no substantial differences in emotional or behavioral problems between children of mothers with vs without breast cancer. Brown et al^9^ found similar results, although they highlighted the benefits of emotional support to children’s psychological adjustment. Such inconsistent findings may be attributed to heterogeneity between studies, such as in sample size, clinical outcome assessment methods, and control group composition.^6,7,8,9,10^

To date, few studies have investigated the emotional problems of children of patients with breast cancer using relevant psychological and clinical scales. Moreover, research into the association of children’s emotional problems and depression with parenting stress of mothers with breast cancer is scarce. Therefore, we aimed to investigate the clinical factors in parenting stress in mothers with breast cancer and the association of maternal depression and parenting stress with their children’s emotional development. Specifically, we examined breast cancer characteristics, treatment, and depression.

## Methods

### Study Design and Participants

This cross-sectional survey included females with stage 0 to 3 breast cancer who visited the breast surgery outpatient clinic at Asan Medical Center in Seoul, South Korea, between June 2020 and April 2021. All participants were aged 20 to 45 years at the time of diagnosis and were currently within 10 years from diagnosis. Patients were excluded from the study if they had (1) recurrent breast cancer; (2) a history of taking psychiatric medications prior to cancer diagnosis, including antipsychotics, antidepressants, and mood stabilizers; (3) an intellectual disability; (4) children with intellectual disabilities, autism spectrum disorders, epilepsy, genetic diseases, or congenital deformities; or (5) children who were not biologically related or older than 12 years. Written informed consent was obtained from all participants. The Asan Medical Center Institutional Review Board approved this cross-sectional study. We followed the Strengthening the Reporting of Observational Studies in Epidemiology (STROBE) reporting guideline.

All participants completed questionnaires that identified their demographic and clinical characteristics as well as assessed their depression. Females with children younger than 12 years also completed rating scales that evaluated their own parenting stress and their children’s emotional and behavioral problems, sleeping habits, and temperament.

### Characteristics and Cancer-Related Factors

Each participant’s demographic and clinical characteristics were collected. These characteristics were age; body mass index; history of any diagnosed disease; familial history of cancer; smoking status; marital status; educational level; spouse’s educational level (only for married participants); children’s ages, any diagnosed disease, and primary caregiver (only for participants with children); and self-reported socioeconomic status (high, middle-high, middle-intermediate, middle-low, and low).

Each participant’s medical records were reviewed to identify various cancer-related factors. These factors were disease duration (defined as the interval between diagnosis and treatment initiation), breast cancer clinical stage, presence of *BRCA1* and *BRCA2* sequence variation, breast surgery type, and other treatment modalities (eg, oophorectomy; radiotherapy [RT]; and systemic treatment, such as chemotherapy, endocrine therapy, or gonadotropin-releasing hormone [GnRH] treatment).

### Clinical Rating Scales

The Center for Epidemiologic Studies Depression–Revised (CESD-R) scale assesses depressive symptoms.^11^ We validated and used the Korean version of the CESD-R scale (score range: 0-60, with the highest score indicating depression).^12^ We considered a cutoff score of 16 or higher as depression. The Korean Parenting Stress Index Short Form (K-PSI-SF) consists of 36 questions, with a 5-point Likert scale grading 3 domains: parental distress, parent-child dysfunctional interaction, and difficult child.^13^ Scores range from 36 to 180, with higher scores indicating greater parenting stress.

The Child Behavior Checklist (CBCL) assesses the emotional and behavioral problems of children and adolescents.^14,15^ A profile of psychological problems can be described using 8 subscales across 3 dimensions of the CBCL: internalizing, externalizing, and total behavioral problems. For these 3 dimensions, the cutoff *T* scores are lower than 60 for the normal range, 60 to 63 for the borderline range, and 64 or higher for the clinical range. For the 8 subscales, the cutoff *T* scores are lower than 65 for the normal range, 65 to 69 for the borderline range, and 70 or higher for the clinical range. A higher CBCL score indicates greater severity of psychosocial problems.

The Junior Temperament and Character Inventory (JTCI) is based on Cloninger’s classification of personality.^16^ It measures 4 different temperament types (novelty seeking, harm avoidance, reward dependence, and persistence) and 3 dimensions of character (self-directedness, cooperativeness, and self-transcendence).^17^

The Children’s Sleep Habits Questionnaire (CSHQ) screens for major medical and behavioral sleep disorders in children.^18^ It comprises 8 subscales: bedtime resistance, sleep duration, parasomnia, sleep-disordered breathing, night wakings, daytime sleepiness, sleep anxiety, and sleep-onset delay. Scores range from 45 to 135, with higher scores indicating sleep disorder.

### Statistical Analysis

The association of mother’s breast cancer with their children’s emotional development was assessed by comparison with reference values. Comparisons between patients with and patients without children were conducted using χ^2^ or Fisher exact tests for categorical variables and unpaired, 2-tailed *t* tests or Wilcoxon rank-sum tests for continuous variables. Analysis of variance and Kruskal-Wallis tests were used to compare CESD-R scale and K-PSI-SF scores according to disease duration. Univariable and multivariable logistic regression analyses were performed to examine the correlates of depression among patients with breast cancer, and a CESD-R scale score of 16 or higher was used as the dependent variable. Variables with *P* < .10 in the univariable analysis were entered into the multivariable analysis using backward elimination. In addition, univariable and multivariable linear regression analyses using stepwise selection were performed with the K-PSI-SF score as the dependent variable to investigate factors associated with level of parenting stress in patients with children. The proportion of participants who scored in the borderline and clinical ranges on the CBCL was calculated. Given the associations between disease duration and depression and between depression and parenting stress, we analyzed the CESD-R scale and K-PSI-SF scores according to disease duration. Thus, disease duration was categorized into 6 categories: less than 1, 1 to 2, 2 to 3, 3 to 4, 4 to 5, and more than 5 years.

A 2-sided *P* < .05 was considered statistically significant. All statistical analyses were performed using SAS, version 9.4 (SAS Institute), and R, version 3.6.1 (R Project for Statistical Computing).

## Results

Of the total 699 females with breast cancer enrolled (mean [SD] age, 39.6 [4.6] years), 499 had children (mean [SD] age of children, 8.0 [2.7] years). Among the 499 patients with children, 490 gave birth before being diagnosed with breast cancer and 9 gave birth after breast cancer treatment. Table 1 shows the demographic and clinical characteristics and cancer-related factors between patients with and without children. Patients without children were younger (mean [SD] age, 37.3 [5.9] vs 40.5 [3.6] years; *P* < .001), had a lower body mass index (calculated as weight in kilograms divided by height in meters squared; 22.3 [3.3] vs 22.9 [3.6]; *P* = .046), and were less likely to have a history of any diagnosed disease (32.5% [65 of 200] vs 43.7% [218 of 499]; *P* = .007) than patients with children. Patients without children were more likely to be smokers (4.0% [8 of 200] vs 0.2% [1 of 499]; *P* < .001) and to have graduated from college (91.5% [183 of 200] vs 84.0% [419 of 499]; *P* = .01). Regarding cancer-related factors, there were no significant differences in disease duration, cancer stage, or *BRCA* sequence variation between patients with and without children. However, a higher proportion of patients without children had breast-conserving surgery (69.5% [139 of 200] vs 50.7% [253 of 499]; *P* < .001) and RT (65.0% [130 of 200] vs 54.3% [271 of 499]; *P* = .005) than patients with children. Other treatment modalities, including oophorectomy, and systemic treatments, such as chemotherapy, endocrine therapy, and GnRH treatment, did not differ between the 2 groups.

**Table 1.** Baseline Characteristics of Patients With Breast Cancer According to Presence of Children

Characteristic	Patient group, No. (%)		*P* value
	With children (n = 499)	Without children (n = 200)	
**Demographic and clinical factors**			
Age, mean (SD), y	40.5 (3.6)	37.3 (5.9)	<.001
BMI, mean (SD)	22.9 (3.6)	22.3 (3.3)	.046
History of any disease diagnosis	218 (43.7)	65 (32.5)	.007
Familial history of cancer	160 (32.1)	73 (36.5)	.26
Smoker	1 (0.2)	8 (4.0)	<.001
Marital status			
Single	0	138 (69.0)	NA
Married	481 (96.3)	61 (30.5)	
Divorced	4 (0.8)	0	
Widowed	0	1 (0.5)	
Educational level			
College graduate	419 (84.0)	183 (91.5)	.01
High school graduate	79 (15.8)	17 (8.5)	
Spouse’s educational level			
College graduate	428 (85.8)	51 (25.5)	NA
High school graduate	70 (14.1)	13 (6.5)	
NA	0	136 (68.0)	
SES			
High	7 (1.4)	3 (1.5)	.98
Middle-high	76 (15.2)	29 (14.5)	
Middle-intermediate	299 (60.0)	121 (60.5)	
Middle-low	100 (20.0)	43 (21.5)	
Low	13 (2.6)	4 (2.0)	
**Cancer-related factors**			
Disease duration, median (IQR), y	1.4 (0.6-3.2)	1.41 (0.7-3.7)	.43
Cancer stage			
0	45 (9.0)	28 (14.0)	.14
1	195 (39.1)	70 (35.0)	
2	190 (38.1)	81 (40.5)	
3	69 (13.8)	21 (10.5)	
*BRCA1* sequence variation	2 (0.4)	2 (1.0)	.32
*BRCA2* sequence variation	9 (1.8)	7 (3.5)	.17
Breast surgery type			
BCS	253 (50.7)	139 (69.5)	<.001
Mastectomy	244 (48.9)	61 (30.5)	
None	2 (0.4)	0	
Oophorectomy	8 (1.6)	0	.11
RT	271 (54.3)	130 (65.0)	.005
Systemic treatment			
Chemotherapy	244 (49.0)	85 (42.5)	.16
Endocrine therapy	381 (76.4)	159 (79.5)	.23
GnRH treatment	230 (46.1)	105 (52.5)	.09

Abbreviations: BCS, breast-conserving surgery; BMI, body mass index (calculated as weight in kilograms divided by height in meters squared); GnRH, gonadotropin-releasing hormone; NA, not available; RT, radiotherapy; SES, socioeconomic status.

Table 2 shows the risk factors for depression among patients with breast cancer. Univariable logistic regression analysis indicated that age (odds ratio [OR], 0.95; 95% CI, 0.92-0.99), smoking (OR, 4.50; 95% CI, 1.19-16.97), high school educational level (OR, 1.74; 95% CI, 1.08-2.79), disease duration (OR, 0.82; 95% CI, 0.74-0.92), RT (OR, 0.72; 95% CI, 0.50-1.03), and GnRH treatment (OR, 1.75; 95% CI, 1.22-2.51) were associated with depression (Table 2). Multivariable analysis showed that having children (OR, 2.25; 95% CI, 1.01-5.05), younger age (OR, 0.96; 95% CI, 0.92-0.99), and high school educational level (OR, 1.76; 95% CI, 1.06-2.94) were associated with depression. Among the cancer-related factors, disease duration (OR, 0.85; 95% CI, 0.76-0.96) and GnRH treatment (OR, 1.68; 95% CI, 1.15-2.44) were associated with depression. Among patients with children, the risk of depression was associated with children’s physical illness (OR, 3.30; 95% CI, 1.29-8.42), mother and other family members being primary caregivers (OR, 1.97; 95% CI, 1.16-3.32), GnRH treatment (OR, 1.93; 95% CI, 1.18-3.14), CSHQ–Sleep-Disordered Breathing score (OR, 1.78; 95% CI, 1.17-2.72), and total K-PSI-SF score (OR, 1.06; 95% CI, 1.04-1.07) (Table 3). Chemotherapy had no significant role in the association between GnRH treatment and depression (eTable in Supplement 1; *P* for interaction = .77).

**Table 2.** Risk Factors for Depression Among Patients With Breast Cancer

Factor	OR (95% CI)	
	Univariable analysis^a^	Multivariable analysis
Patient group		
Without children	1 [Reference]	1 [Reference]
With children	1.98 (0.91-4.27)	2.25 (1.01-5.05)
Single	2.21 (0.96-5.10)	2.11 (0.89-4.98)
Age, y	0.95 (0.92-0.99)	0.96 (0.92-0.99)
BMI	1.00 (0.95-1.05)	NA
History of any disease diagnosis	1.41 (0.99-2.02)	1.46 (0.99-2.12)
Familial history of cancer	1.26 (0.87-1.83)	NA
Smoker	4.50 (1.19-16.97)	4.50 (1.01-20.08)
Married	0.83 (0.54-1.28)	NA
Educational level		
College graduate	1 [Reference]	1 [Reference]
High school graduate	1.74 (1.08-2.79)	1.76 (1.06-2.94)
Spouse’s educational level		
College graduate	1 [Reference]	NA
High school graduate	1.47 (0.87-2.50)	NA
NA	1.23 (0.78-1.93)	NA
SES		
High	1 [Reference]	NA
Middle-high	0.88 (0.17-4.50)	NA
Middle-intermediate	1.06 (0.22-5.08)	NA
Middle-low	1.66 (0.34-8.16)	NA
Low	0.53 (0.06-4.53)	NA
Disease duration, y	0.82 (0.74-0.92)	0.85 (0.76-0.96)
Cancer stage		
0	1 [Reference]	NA
1	2.04 (0.99-4.22)	NA
2	1.75 (0.85-3.63)	NA
3	1.92 (0.84-4.38)	NA
*BRCA1* or *BRCA2* sequence variation	0.87 (0.29-2.65)	NA
Breast surgery type		
BCS	1 [Reference]	NA
Mastectomy	1.02 (0.71-1.46)	NA
None	3.56 (0.22-57.48)	NA
Oophorectomy	1.17 (0.23-5.87)	NA
RT	0.72 (0.50-1.03)	NA
Chemotherapy	0.91 (0.63-1.30)	NA
Endocrine therapy	0.74 (0.49-1.11)	NA
GnRH treatment	1.75 (1.22-2.51)	1.68 (1.15-2.44)

Abbreviations: BCS, breast-conserving surgery; BMI, body mass index; GnRH, gonadotropin-releasing hormone; NA, not available; OR, odds ratio; RT, radiotherapy; SES, socioeconomic status.

^a^ Variables with a *P* < .10 in the univariable analysis were entered into the multivariable analysis using backward elimination.

**Table 3.** Risk Factors for Depression Among Mothers With Breast Cancer

Factor	OR (95% CI)	
	Univariable analysis	Multivariable analysis^a^
Children’s physical illness	2.34 (1.06-5.16)	3.30 (1.29-8.42)
Primary caregiver status		
Mother	1 [Reference]	1 [Reference]
Mother and other family members	1.37 (0.88-2.14)	1.97 (1.16-3.32)
Other family members	0.62 (0.28-1.39)	0.65 (0.27-1.57)
Disease duration, y	0.83 (0.73-0.95)	0.86 (0.74-1.00)
GnRH treatment	1.90 (1.24-2.91)	1.93 (1.18-3.14)
CSHQ–Sleep Duration	1.29 (1.10-1.50)	1.17 (0.98-1.40)
CSHQ–Sleep-Disordered Breathing	1.81 (1.25-2.62)	1.78 (1.17-2.72)
Total K-PSI-SF score	1.06 (1.04-1.07)	1.06 (1.04-1.07)

Abbreviations: CSHQ, Children’s Sleep Habits Questionnaire; GnRH, gonadotropin-releasing hormone; K-PSI-SF, Korean Parenting Stress Index Short Form; OR, odds ratio.

^a^ All 499 patients were included in the multivariable logistic regression analysis.

As shown in Table 4, parenting stress was significantly higher in mothers with children 6 years or older (β = 3.09; 95% CI, 0.19-5.99) than in those with younger children (1.5 to <6 years) as well as in mothers who were the sole primary caregiver rather than in those who shared the role with other family members (β = −3.43; 95% CI, −5.87 to −0.99). The JTCI-measured novelty seeking (β = 0.58; 95% CI, 0.46-0.71) and harm avoidance (β = 0.21; 95% CI, 0.09-0.33) temperament types were directly correlated with the K-PSI-SF score, whereas the reward dependence temperament was inversely correlated with the K-PSI-SF score (β = −0.31; 95% CI, −0.43 to −0.19). The anxious/depressed (β = 8.09; 95% ci, 3.34-12.83), attention problem (β = 6.78; 95% ci, 1.52-12.04), and rule-breaking behavior (β = 7.28; 95% ci, 1.67-12.89) CBCL subscale scores of 65 or higher were directly associated with the K-PSI-SF score. The bedtime resistance (β = 0.57; 95% CI, 0.15-0.99), sleep-onset delay (β = 2.04; 95% ci, 0.19-3.89), and daytime sleepiness (β = 0.72; 95% CI, 0.35-1.09) subscale scores of the CSHQ were also directly correlated with the K-PSI-SF score. Similarly, the total CESD-R scale score was correlated with the K-PSI-SF score (β = 0.56; 95% CI, 0.45-0.66). Other cancer-related factors were not associated with the K-PSI-SF score.

**Table 4.** Multivariable Linear Regression Analysis of the Korean Parenting Stress Index Short Form (K-PSI-SF) Scores

Factor	β (95% CI)	
	Univariable analysis	Multivariable analysis
Child’s age, y		
1.5 to <6	1 [Reference]	1 [Reference]
≥6	−0.34 (−4.37 to 3.68)	3.09 (0.19 to 5.99)
Primary caregiver		
Mother	1 [Reference]	1 [Reference]
Mother and other family members	−3.19 (−6.60 to 0.22)	−3.43 (−5.87 to −0.99)
Other family members	−1.84 (−6.99 to 3.32)	−3.20 (−6.86 to 0.45)
JTCI–novelty seeking	0.90 (0.76 to 1.04)	0.58 (0.46 to 0.71)
JTCI–harm avoidance	0.65 (0.50 to 0.79)	0.21 (0.09 to 0.33)
JTCI–reward dependence	−0.23 (−0.39 to −0.06)	−0.31 (−0.43 to −0.19)
CBCL–anxious/depressed, score ≥65	23.21 (17.20 to 29.22)	8.09 (3.34 to 12.83)
CBCL–attention problems, score ≥65	21.45 (14.43 to 28.47)	6.78 (1.52 to 12.04)
CBCL–rule-breaking behavior, score ≥65	21.96 (14.45 to 29.5)	7.28 (1.67 to 12.89)
CSHQ–bedtime resistance	1.57 (1.03 to 2.11)	0.57 (0.15 to 0.99)
CSHQ–sleep-onset delay	5.64 (3.05 to 8.23)	2.04 (0.19 to 3.89)
CSHQ–daytime sleepiness	1.90 (1.42 to 2.38)	0.72 (0.35 to 1.09)
Total CESD-R scale score	0.79 (0.66 to 0.92)	0.56 (0.45 to 0.66)

Abbreviations: CBCL, Child Behavior Checklist; CESD-R, Center for Epidemiologic Studies Depression–Revised; CSHQ, Children’s Sleep Habits Questionnaire; JTCI, Junior Temperament and Character Inventory.

Disease duration was associated with the CESD-R scale score (Figure, A). Despite the decreasing score pattern similar to that of the CESD-R scale score, the K-PSI-SF score was not associated with CESD-R scale score throughout the disease duration (Figure, B).

**Figure.**
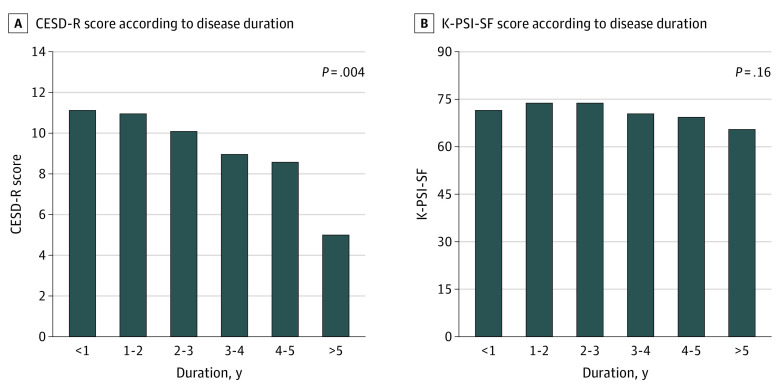
Center for Epidemiologic Studies Depression–Revised (CESD-R) Scale and Korean Parenting Stress Index Short Form (K-PSI-SF) Scores According to Disease Duration Scores for the CESD-R range from 0 to 60, with the highest score indicating depression. Scores for the K-PSI-SF range from 36 to 180, with higher scores indicating greater parenting stress.

Children of patients with breast cancer did not score higher than the normal score ranges for internalizing, externalizing, and total behavioral problem (*T* scores: <64 [92nd percentile] and <60 [84th percentile]) subscales of the CBCL (eFigure in Supplement 1). However, more children of patients with breast cancer scored higher on the anxious/depressed (*T* score: ≥70 [98th percentile]) and thought problems (*T* score: ≥65 [93rd percentile]) subscales than expected based on the CBCL normal ranges.

## Discussion

In this cross-sectional study, we found that parenting stress was associated with depression, and various child-related factors played a role in this association, such as age, temperament, emotional problems, and sleeping patterns. We did not observe an association between the characteristics of breast cancer itself and parenting stress. Having children, a high school educational level, and GnRH treatment were associated with an increased risk of depression among patients with breast cancer. Conversely, older maternal age and a longer disease duration were correlated with a lower risk of depression. Additionally, we found that maternal breast cancer diagnosis and treatment did not have an association with the emotional development of children.

Among cancer-related factors, disease duration and GnRH treatment were associated with maternal depression. Other cancer-related factors, such as breast cancer stage, *BRCA1* and *BRCA2* sequence variation, and other treatment modalities, were not associated with depression, which is consistent with the findings of previous studies investigating the association between depression and breast cancer.^19,20,21^ In line with previous research,^22,23,24^ we found that younger age, having a child, and a lower educational level were all independently correlated with depression. In addition, a short disease duration and GnRH treatment were associated with depression, both of which have previously been reported as risk factors for depression in patients with breast cancer.^25,26^

We found that GnRH treatment was associated with the risk of depression, whereas chemotherapy had no association. Although adverse psychological outcomes of chemotherapy have been previously reported,^27^ the present study highlighted the importance of assessing mental status in those undergoing GnRH treatment, regardless of previous chemotherapy. Brunt and colleagues^28^ reported the substantial role of additional ovarian suppression treatment, which was associated with worsened depression and anxiety induced by chemotherapy. However, we found that GnRH treatment alone increased the risk of depression in patients who had not undergone chemotherapy. In general, patients are educated and prepared for chemotherapy prior to treatment, particularly regarding its potential toxic effects; however, such preparation is not common for endocrine therapy.^29^ This difference may contribute to distress in patients undergoing endocrine therapy.

We found that maternal depression and parenting stress were correlated. Other studies have also reported an association between maternal depression and parenting stress in various clinical samples.^30,31,32^ Given the high prevalence of depression among patients with breast cancer,^33^ active surveillance of depression and timely intervention are recommended to reduce psychological distress. In addition, early intervention for mental health issues may be required for children if their mothers with breast cancer are at greater risk of depression.

Results of the present study also revealed that maternal parenting stress was correlated with maternal primary caregiver status and child behavioral problems, sleeping problems, and temperament but was not associated with cancer-related factors. In contrast to previous studies suggesting that maternal parenting stress was highest for mothers with preschool-aged children,^34^ we found that patients with children 6 years or older reported higher parenting stress. This finding may be attributed to participants’ limited ability (given their diagnosis and/or treatment) to help their children adjust to school activities. Mothers who were the sole caregiver had significantly higher parenting stress than those who shared caregiving with other family members. Although the presence of additional caregivers was associated with reduced parenting stress, it was also associated with increased risk of depression, which may be attributed to the mother’s relationship with the other caregivers, such as parents or parents-in-law (children’s grandparents). However, we did not analyze these factors because such details were unavailable.

Novelty seeking and harm avoidance in children were directly correlated, whereas reward dependence was inversely correlated, with parenting stress.^35^ Novelty seeking represents curiosity and impulsivity, harm avoidance represents anxiousness, and reward dependence represents social contact and devotion.^36^ We found that children’s poor sleeping habits (bedtime resistance, sleep-onset delay, and daytime sleepiness) and behavioral problems (anxiety, depression, attention problems, and rule-breaking behaviors) were directly associated with the K-PSI-SF score. Among all clinical variables, these associations were most prominent for the CBCL measures. This finding is consistent with previous research that suggested that child caretaking responsibility^37^ and internalizing and externalizing behaviors of children may be factors in higher parenting stress, although this association may be bidirectional.^38^ Results of the present study indicated that parenting stress may be more likely to be affected by child-related factors rather than cancer- or treatment-related factors. Thus, when treating mothers with breast cancer, clinicians should pay attention to the children’s mental health and parenting conditions.

Among children of mothers with breast cancer, no remarkable association was noted between child emotional development and maternal breast cancer diagnosis or treatment. Studies investigating the emotional development of these children have reported inconsistent results. John et al^39^ reported that children of mothers with breast cancer did not adjust as well as children in the general population in terms of overall quality of life and psychopathological symptoms. In contrast, Armsden and Lewis^7^ found that children of mothers with breast cancer had significantly fewer emotional or social problems compared with normative data.^7^ Billhult et al^40^ observed that mothers with breast cancer tried to maintain a routine of daily activities for their children. Furthermore, Asbury and colleagues^41^ found that patients with breast cancer tended to conceal their depressive mood to protect their children emotionally.

A meta-analysis revealed that maternal depression had implications for children’s emotional development.^42^ Therefore, interventions for depression and parenting stress may be beneficial for both patients with breast cancer and their children.^43^ One study reported that mothers’ concealment of their breast cancer did not benefit the children.^41^ Moreover, maternal diagnosis played a role in adolescent children’s susceptibility to experiencing emotional stress.^44^ Therefore, interventions to build solid relationships between patients and their children are crucial.^38,45,46^

The results of this study suggest the importance of identifying high-risk population and psychological intervention to relieve the parenting stress of patients with breast cancer. Further studies are warranted to evaluate the effectiveness of psychological consultation or medication to reduce parenting stress. In addition, because of the cross-sectional study design, it is imperative to evaluate in a separate longitudinal study the implications of maternal breast cancer for children as they continue to mature.

### Strengths and Limitations

The study has several strengths and limitations. First, there was an imbalance between groups (patients with vs without children) in the number of participants, demographic characteristics, and treatments. Second, this single-center study may limit the generalizability of the results. However, participants received standardized treatment and surveillance. In addition, there was a large sample size and validated questionnaires were used, which are strengths of the study. Third, we did not include children of mothers without breast cancer. Instead, we compared the CBCL profiles of the children of patients with breast cancer with population-based, age- and sex-adjusted normal scores. Fourth, as an inherent feature of a cross-sectional design, this study could not draw a conclusion on potential factors or the directionality of associations between variables.

## Conclusions

This cross-sectional study found that among mothers with breast cancer, parenting stress was associated with child-related factors and depression, while no associations were found with breast cancer–related factors. Within this patient population, having children, short disease duration, and GnRH treatment were identified as risk factors for depression. These findings underscored the susceptibility of mothers with breast cancer to both depression and parenting stress, regardless of disease duration, emphasizing the imperative for counseling and support tailored to this patient group. Furthermore, this research highlighted that emotional development of children whose mothers had breast cancer was not significantly different from reference values, which could offer solace to these patients amid the challenges they encounter during their illness.

## Notes

Supplement 1. **eTable.** Odds Ratio of Gonadotropin-Releasing Hormone Agonist Treatment on the Center for Epidemiologic Studies Depression Score According to Addition of Chemotherapy **eFigure.** Distribution of the Child Behavior Checklist (CBCL) Scores of Children of Breast Cancer Patients

Supplement 2.
Data Sharing Statement
