# Supplementary material for: Factors in Parenting Stress in Young Patients With Breast Cancer and Implications for Children’s Emotional Development: The PSYCHE Study
Source: JAMA Netw Open. 2023 Nov 28;6(11):e2344835. doi: 10.1001/jamanetworkopen.2023.44835 (PMC10685886; doi:10.1001/jamanetworkopen.2023.44835)
Supplement: Supplement 1. — eTable. Odds Ratio of Gonadotropin-Releasing Hormone Agonist Treatment on the Center for Epidemiologic Studies Depression Score According to Addition of Chemotherapy eFigure. Distribution of the Child Behavior Checklist (CBCL) Scores of Children of Breast Cancer Patients [file jamanetwopen-e2344835-s001.pdf]

Supplemental Online Content

Shin Y, Kim H, Lee T, et al. Factors in parenting stress in young patients with breast cancer and implications for children's emotional development: the PSYCHE study. *JAMA Netw Open*. 2023;6(11):e2344835. doi:10.1001/jamanetworkopen.2023.44835

eTable. Odds Ratio of Gonadotropin-Releasing Hormone Agonist Treatment on the Center for Epidemiologic Studies Depression Score According to Addition of Chemotherapy

eFigure. Distribution of the Child Behavior Checklist (CBCL) Scores of Children of Breast Cancer Patients

This supplemental material has been provided by the authors to give readers additional information about their work.

© 2023 Shin Y et al. *JAMA Network Open*

eTable. Odds ratio of gonadotropin-releasing hormone agonist treatment on the Center for Epidemiologic Studies Depression score according to addition of chemotherapy

				Univariate					Multivariable				
Variable		GnRHa (event/N) No	GnRHa (event/N) Yes	OR	95% CI		*P*-value	*P*-value of the interaction	OR	95% CI		*P*-value	*P*-value of the interaction
Chemo therapy	No	29/144	31/111	1.54	0.86	2.75	.147	.278	1.90	0.97	3.72	.062	.769
	Yes	18/125	35/119	2.48	1.31	4.68	.005		2.20	1.06	4.57	.035	

A total of 499 patients with children were included in the multivariable logistic regression analysis.

Children's physical illness, primary caregiver status, past medical history, disease duration (year), CSHQ-SD score, CSHQ-SDB score, K-PSI-SF score, GnRHa use, chemotherapy, and the interaction between GnRHa and chemotherapy were included in the multivariable logistic regression analysis.

CI = confidence interval; CSHQ = Children's Sleep Habits Questionnaire; GnRHa = gonadotropin-releasing hormone agonist; JTCI = Junior Temperament and Character Inventory; OR = odds ratio; SD = Sleep Duration; SDB = Sleep Disordered Breathing; K-PSI-SF = Parenting Stress Index

© 2023 Shin Y et al. *JAMA Network Open*

eFigure. Distribution of the Child Behavior Checklist (CBCL) scores of children of breast cancer patients

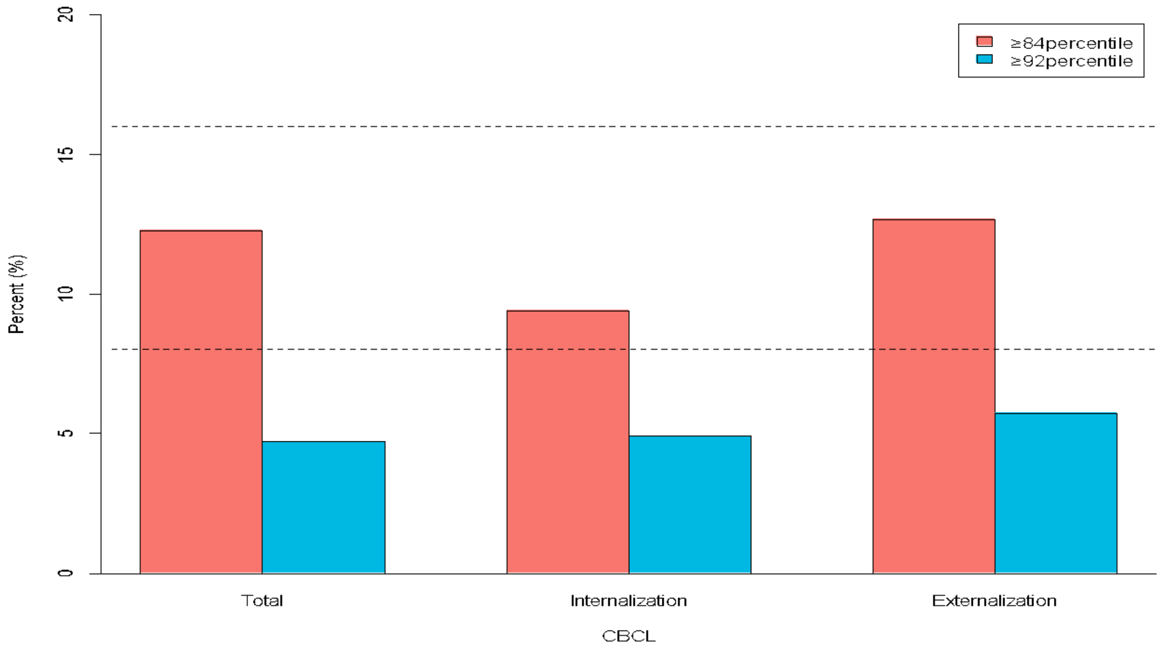

© 2023 Shin Y et al. *JAMA Network Open*

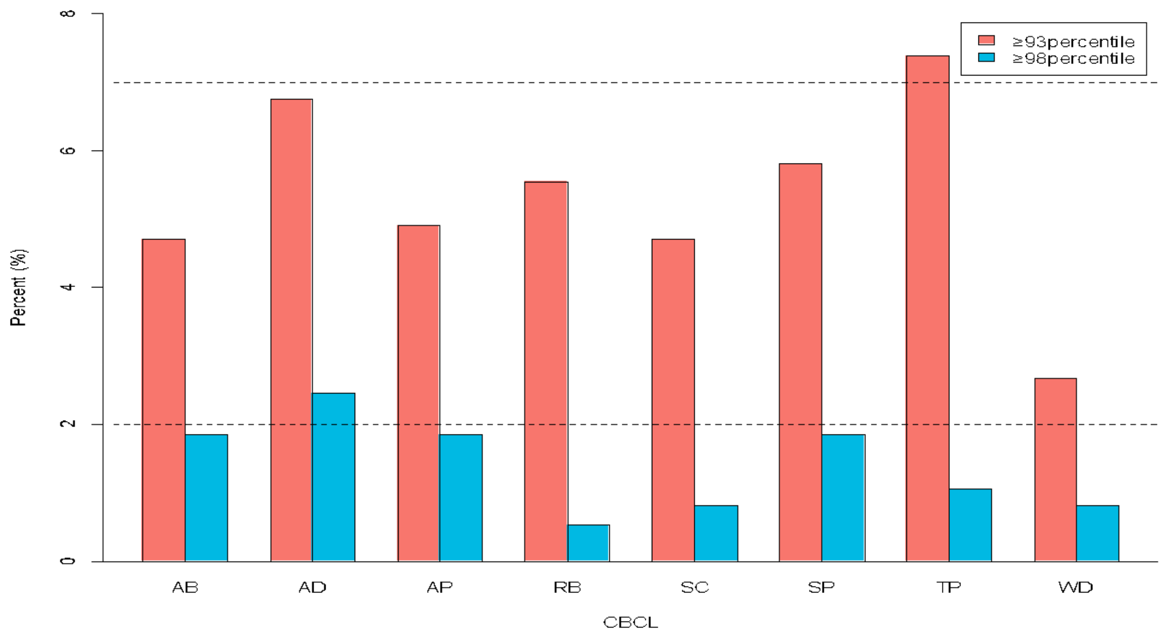

AB = Aggressive Behaviors; AD = Anxiety/Depressed; AP = Attention Problems; CBCL = Child Behavior Checklist; RB = Rule-breaking Behaviors; SC = Somatic Complaints; SP = Social Problems; TP = Thought Problems; WD = Withdrawn/Depressed.

© 2023 Shin Y et al. *JAMA Network Open*
