# Supplementary material for: Factors in Parenting Stress in Young Patients With Breast Cancer and Implications for Children’s Emotional Development: The PSYCHE Study
Source: JAMA Netw Open. 2023 Nov 28;6(11):e2344835. doi: 10.1001/jamanetworkopen.2023.44835 (PMC10685886; doi:10.1001/jamanetworkopen.2023.44835)
Supplement: Supplement 2. — Data Sharing Statement [file jamanetwopen-e2344835-s002.pdf]

Data Sharing Statement

Shin. Factors in Parenting Stress in Young Patients With Breast Cancer and Implications for Children’s Emotional Development. *JAMA Netw Open*. Published November 28, 2023. doi:10.1001/jamanetworkopen.2023.44835

Data
Data available: No
